# Lynch syndrome screening in colorectal and endometrial cancers in Iceland

**DOI:** 10.2340/1651-226X.2025.41957

**Published:** 2025-01-31

**Authors:** Katla R. Kluvers, Thordur Tryggvason, Vigdis Stefansdottir, Jon G. Jonasson, Petur Snaebjornsson, Sigurdis Haraldsdottir

**Affiliations:** a Faculty of Medicine, University of Iceland, Reykjavik, Iceland; b Department of Pathology, Landspitali University Hospital, Reykjavik, Iceland; c Department of Genetics and Molecular Medicine, Landspitali University Hospital, Reykjavik, Iceland; d Department of Pathology, Netherlands Cancer Institute, Amsterdam, the Netherlands; e Department of Oncology, Landspitali University Hospital, Reykjavik, Iceland

**Keywords:** Lynch syndrome, mismatch repair deficiency, MMR, immunohistochemistry, founder variant, cancer genetics

## Abstract

**Background and purpose:**

Screening for Lynch syndrome (LS) with mismatch repair (MMR) protein immunohistochemistry (IHC) in all patients with newly diagnosed colorectal (CRC) and endometrial cancer (EC) was implemented in Iceland in 2017. The aim of the study is to assess the accuracy of screening in 2020–2022 and compare it to 2017–2019 when screening was initiated.

**Patients/materials and methods:**

All patients diagnosed with CRC and EC according to the Icelandic Cancer Registry in 2020–2022 were included. Screening results were crossmatched with a genotyping database from deCODE to calculate sensitivity and specificity for LS detection.

**Results:**

In 2020–2022, 429 of 522 (82%) diagnosed CRCs were stained and 90 of 106 (85%) ECs, compared to 74% of CRCs and 82% of ECs in 2017–2019. The screening protocol was followed in 90% of cases for CRCs and 95% of cases for ECs compared to 89% and 68% during 2017–2019. The sensitivity of IHC as a screening method for LS was 70% and specificity 88% with a positive and negative predictive value of 8.4% and 99.4%, respectively.

**Interpretation:**

Three LS cases were missed with MMR IHC (1 *MSH6* and 2 *PMS2* carriers), it is possible these patients had sporadic cancers unrelated to their LS carrier status. MSH6 and PMS2 deficiency strongly predicts LS in Iceland.

## Introduction

Lynch syndrome (LS) is a hereditary cancer syndrome caused by pathogenic germline variants in one of the four mismatch repair (MMR) genes: *MLH1, MSH2, MSH6, PMS2* or *EPCAM* [1]*.* LS is the most common hereditary cause of colorectal cancer (CRC) and increases the lifetime risk of multiple cancer types, most notably colorectal and endometrial cancer (EC) [2]. LS has a documented population prevalence from 1:370 to 1:2000 [3] and has been reported to be as high as 1:226 in the Icelandic population [4]. This is in part because of a high prevalence of *MSH6* and *PMS2* founder variants in Iceland [4]. A hallmark of LS tumors is MMR deficiency (dMMR) with absent staining of corresponding MMR proteins on immunohistochemistry (IHC) on tumor tissue, which can be used as a screening method for LS [1]. Up to 15% of all diagnosed CRCs and 20% of all ECs are MMR deficient, but only about 2–3% of CRCs and ECs carry LS [1, 5].

Screening for LS with MMR IHC in all newly diagnosed CRCs and ECs was implemented universally in Iceland in 2017. We previously published results on the efficacy of IHC screening for LS in 2017–2019 where screening accurately identified 89% of LS cases [6]. The aim of this study is to assess the efficacy of screening in 2020–2022 and compare to 2017–2019 when screening was initiated.

All patients diagnosed with CRC or EC in 2020–2022 were included in this retrospective cohort study. The Icelandic Cancer Registry captures all cancer diagnoses in the country and has an accuracy of 99.15% [7]. All tumor MMR IHC and clinical genetic testing is performed centrally at Landspitali University Hospital; clinical genetic testing is performed with Illumina TruSight Hereditary Cancer Panel. deCODE Genetics have collected extensive genotyping information on the Icelandic population as previously described [8] with whole-genome sequencing performed on 49,708 Icelanders using Illumina technique and germline variants have been imputed into 116,573 Icelanders whose DNA has been genotyped with Illumina single nucleotide polymorphism chips and phased using long-range phasing [9]. Written informed consent was obtained from all genotyped subjects. Variants in *MLH1*, *MSH2* (*EPCAM*), *MSH6* and *PMS2* for the study subjects with available genotypes were extracted from deCODE’s genotyping database and were crossmatched with the screening protocol to calculate the sensitivity and specificity of the screening method for LS. The screening protocol was considered to be correctly followed if the algorithm was followed until a diagnosis of *MLH1*-hypermethylation (where all appropriate *BRAF* and *MLH1*-hm testing had been done) or LS.

## Results and discussion

A total of 522 colorectal adenocarcinoma cases and 106 endometrial carcinoma cases were diagnosed in 2020–2022 according to the Icelandic Cancer Registry. In 2020–2022, 429 of 522 (82%) CRC cases were stained and 90 of 106 (85%) EC cases as compared to 74% of CRCs and 82% of ECs during screening in 2017–2019 [6].

Out of 429 stained CRC specimens, 63 (15%) were MMR deficient and out of 90 stained EC specimens, 20 (22%) were dMMR. Table 1 shows the staining pattern loss in dMMR tumors as well as the etiology of dMMR status. The screening protocol was correctly followed in 90% of CRC cases and 95% of EC cases. Referral for genetic counseling and germline testing was correctly made in all dMMR cases, which was an improvement from the 2017–2019 period where only 50% were appropriately referred for clinical genetic testing. A list of all stained CRCs and ECs was crossmatched with deCODE’s genotyping database, where 535 cases altogether had available data at deCODE. A total of 10 CRC or EC cases were identified to carry LS, of which 7 LS cases were identified through the clinical universal screening protocol at Landspitali University Hospital. One known LS carrier with a *MSH6* founder variant had normal MMR IHC on CRC and would therefore not have been found by MMR IHC screening and two LS carriers with a *PMS2* founder variant (not known to the genetics clinic) had normal MMR IHC on CRC. Therefore, three LS cases did not present with absence of MMR proteins on IHC staining and were missed during screening. The sensitivity of IHC as a screening method for LS was 70%, the specificity 87.7%, the positive predictive value 8.4% and the negative predictive value 99.4%. LS was found in 10 out of 535 (1.9%) cases, which corresponds to LS prevalence estimates of 2–3% in CRC and EC studies [1, 5]. Five of 10 (50%) patients with LS had a pathogenic germline variant in *MSH6* and three of 10 (30%) had a pathogenic variant in *PMS2* and a single patient carried a *MLH1* translocation, all of which have been previously described in Iceland [4]. One patient carried a *MSH2-EPCAM* deletion not previously described in Iceland. The absence of MSH6 or PMS2 alone strongly predicts LS as 100% of these cases were found to carry LS and corresponds to previously described results in cohorts from Iceland and Ohio [10].

**Table 1:** Etiology of MMR deficiency in colorectal and endometrial cancers diagnosed 2020–2022.

	MLH1-hm*	Lynch sx	Unexplained**	Total
**Colorectal cancer *n* = 63**				
MLH1/PMS2 or MLH1*** missing	52	1	6	59
MSH2/MSH6 missing		1		1
MSH6 missing		1		1
PMS2 missing		1		1
All 4 stains missing				1****
**Endometrial cancer *n* = 20**				
MLH1/PMS2 or MLH1*** missing	15	0	1	16
MSH2/MSH6 missing		1	1	2
MSH6 missing		2		2
PMS2 missing		0		0

MLH1-hm: *MLH1* hypermethylation; Lynch sx: Lynch syndrome.

* Reflex testing with *BRAF* mutational analysis in colorectal cancer followed by *MLH1* methylation testing if *BRAF* wildtype. Reflex testing in endometrial cancer with *MLH1* methylation testing.

** In 5 of 6 colorectal cases *BRAF* or *MLH1*-hm testing was not done, in one case it was normal and germline testing was negative. In the endometrial case with MLH1/PMS2 deficiency, *MLH1*-hm testing was not done. In the MSH2/MSH6 deficient case, germline testing was normal and tumor testing was not done.

*** Six colorectal cases had weak PMS2 staining with MLH1 missing and 5 were *MLH1*-hm (one was not tested). One endometrial case had weak PMS2 staining with MLH1 missing and was *MLH1*-hm.

**** MMR immunohistochemistry was repeated and was normal.

We found that 3 of 10 (30%) cases of LS had been missed during screening because they did not present with absent staining of any MMR protein but one of these cases was already known to the genetics clinic. It is quite possible that the *PMS2* carriers developed sporadic CRCs, not caused by LS. The MSH6 MMR IHC can be difficult to interpret and it is not clear if the *MSH6* carrier developed a sporadic tumor or that the MMR IHC was not representative of MMR deficiency. Screening for LS with IHC is reflexive and as noted earlier, the Icelandic population has higher prevalence of founder variants in the *MSH6* and *PMS2* genes, causing a less striking phenotype than seen in LS caused by variants in the *MLH1* and *MSH2* genes. Our study suggests that MMR IHC is less reliable as a screening test in such a population.

## Data Availability

The de-identified data that support the findings of this study are available on request from the corresponding author.
